# A pathogenic *progranulin* mutation and *C9orf72* repeat expansion in a family with frontotemporal dementia

**DOI:** 10.1111/nan.12100

**Published:** 2014-04-22

**Authors:** Tammaryn Lashley, Jonathan D Rohrer, Colin Mahoney, Elizabeth Gordon, Jon Beck, Simon Mead, Jason Warren, Martin Rossor, Tamas Revesz

**Affiliations:** * Queen Square Brain Bank, Department of Molecular Neuroscience, UCL Institute of NeurologyLondon, UK; † Dementia Research Centre, UCL Institute of NeurologyLondon, UK; ‡ MRC Prion Unit, Department of Neurodegenerative Diseases, UCL Institute of NeurologyLondon, UK

**Keywords:** *C9orf72*, FTLD, progranulin, TDP-43

## Abstract

**Aims:**

Frontotemporal lobar degeneration (FTLD) is a progressive neurodegenerative disease and is the second most common form of young onset dementia after Alzheimer's disease (AD). An autosomal dominant pattern of inheritance is present in around 25–50% of FTLD cases indicating a strong genetic component. Major pathogenic mutations of FTLD have been demonstrated independently in the progranulin (*GRN*) gene and the *C9orf72* hexanucleotide expansion repeat. In this study we present a family that have been identified as carrying both a *GRN Cys31fs* mutation and the *C9orf72* hexanucleotide expansion repeat.

**Methods:**

In the present study we describe the clinical and genetic details of family members and pathological features of two family members that have come to post-mortem.

**Results:**

The mean age at disease onset was 57 years (48–61 years) and mean duration 4 years (2–7 years). The most common presenting syndrome was behavioural variant frontotemporal dementia. Brain imaging from available cases showed a symmetrical pattern of atrophy particularly affecting the frontal and temporal lobes. Pathologically two cases were classified as FTLD-TDP type A with TDP-43 positive inclusions, with additional p62-positive ‘star-like’ inclusions found in the hippocampal formation and cerebellum.

**Conclusions:**

The type and distribution of the pathological lesions in these two cases were in keeping with FTLD cases carrying only the *C9orf72* hexanucleotide repeat. However the driving force of the pathological process may be either pathogenic mutation or a combination of both converging on a singular mechanism.

## Introduction

Frontotemporal lobar degeneration (FTLD) is a progressive neurodegenerative disease and is the second most common form of dementia with presenile onset after Alzheimer's disease (AD). Clinically, there are three major syndromes, behavioural variant frontotemporal dementia (bvFTD), semantic dementia (SD) and progressive non-fluent aphasia (PNFA), whilst pathologically, the majority of cases have inclusions containing either tau (FTLD-tau) or TDP-43 (FTLD-TDP). A family history of FTLD, often showing an autosomal dominant pattern of inheritance is present in around 25–50% of cases indicating a strong genetic component [1],[2]. Major genetic causes of FTLD include mutations in the *MAPT* [3] and progranulin (*GRN*) genes [4] both located on chromosome 17. Recently, two independent studies identified a GGGGCC hexanucleotide expansion in the chromosome 9 open reading frame 72 (*C9orf72*) gene as a further common genetic cause of FTLD [5],[6]. The clinical phenotype of individuals with a *GRN* mutation is variable with patients presenting with bvFTD, PNFA or in some cases, a corticobasal syndrome [7]. The first *GRN* mutation associated with FTLD was identified in 2006. Since then over 70 different *GRN* mutations have been reported in more than 230 families worldwide, which accounts for 5–20% of cases of familial FTD and 1–5% of sporadic cases [8]. Most mutations are predicted to result in premature termination codons and it has been shown that the mutant mRNA is rapidly degraded through the process of nonsense mediated decay, resulting in a functional null allele [4]. All cases with known *GRN* mutations are associated with underlying FTLD-TDP type A pathology, characterized by neuronal cytoplasmic inclusions (NCIs), occasional neuronal intranuclear inclusions (NIIs) and short neurites.

*C9orf72* expansions are associated with a heterogeneous clinical presentation and are highly variable between and within family members [9]. Between 7% and 12% of all FTD cohorts are found to have the mutation 9–11. Patients can present with FTD, ALS or mimic several other neurodegenerative disease syndromes [12]. The FTD subtype is most often bvFTD with PNFA being observed occasionally. ALS typically shows early involvement of both upper and lower motor neurones and bulbar presentation is common 13–18. The neuropathology associated with the *C9orf72* expansion repeat combines both FTLD-TDP and ALS. Previous mutations associated with FTLD, have fallen neatly into one of the pathologically determined subtypes; *GRN* with FTLD-TDP type A and *VCP* with FTLD-TDP type D. However, the *C9orf72* expansion repeat crosses the pathological boundaries and has been shown to be associated with two FTLD-TDP subtypes; type A and B [9]. Cases with this expansion also show unique pathology in addition to the TDP-43 inclusions with TDP-43 negative, p62-positive NCIs found predominantly in the hippocampal formation and cerebellar granule cell layer [9],[19].

In the present study we present a family that have been identified as carrying both a *GRN Cys31fs (g.90_91insCTGC)* pathogenic mutation in addition to the *C9orf72* hexanucleotide expansion repeat. We describe the clinical, genetic and pathological features of two of the family members that have now come to post-mortem. We compare the clinical and pathological findings with FTLD cases affected by either a *GRN* or a *C9orf72* hexanucleotide expansion and discuss the relevance of both mutations, speculating which may be driving the pathogenic mechanism.

## Material and methods

### Cases

The two reported cases donated their brains to the Queen Square Brain Bank but had clinically been seen in the Dementia Research Centre (UCL), they underwent a standard clinical history, neurological examination and cognitive assessment. Ethical approval for the study was obtained from the National Hospital for Neurology and Neurosurgery Local Research Ethics Committee.

### Genetics

DNA extraction, Rs3849942 genotyping, hexanucleotide repeat number assessment, DNA sequencing and Southern blotting were carried out as described previously [5],[6],[12].

### Assessment of brain volume

We assessed this in a formal analysis of the volume difference between the right and left hemispheres in a group of 15 patients with *C9orf72* expansions [10 men, 5 women, mean (standard deviation) age 59.5 (7.8)], 10 patients with *GRN* mutations [6 men, 4 women, 61.0 (7.0)] and 15 cognitively normal controls [10 men, 5 women, 57.7 (5.3)] using the following methods [20]: a whole-brain region was created by segmenting the brain using a semi-automated technique in the MIDAS software package [21]. Scans and associated whole-brain regions were then transformed into standard space by registration to the Montreal Neurological Institute (MNI) Template. The left and right hemispheric regions were defined using the MNI average brain which was split by dividing the whole volume along a line coincident with the interhemispheric fissure. An intersection of each individual's brain region and the hemispheric regions defined on the MNI template was generated to provide a measure of brain volume in left and right hemispheres, with the difference between the two hemispheres calculated by subtracting one from the other.

### Immunohistochemistry

Brains were routinely dissected and blocks removed according to the Queen Square Brain Bank protocol. Seven micron thick tissue sections were immunostained using commercially available antibodies to the following proteins: TDP-43 (Abnova, Taipei City, Taiwan; 1:800); p62 (BD Transduction Labs, Oxford, UK; 1:200); ubiquitin (Dako, Ely, UK; 1:200); ubiquilin2 (Abnova, Taipei City, Taiwan; 1:1000); α-synuclein (Vector, Peterborough, UK; 1:50), tau (AT8 clone; Autogen Bioclear, Wiltshire, UK; 1:600); CD68 (Dako, Ely, UK; 1:150), GFAP (Dako, Ely, UK; 1:1000) as previously described [22]. Briefly, immunohistochemistry for all antibodies required pressure cooker pre-treatment in citrate buffer pH 6.0. Endogenous peroxidase activity was blocked with 0.3% H_2_0_2_ in methanol and non-specific binding with 10% dried milk solution. Tissue sections were incubated with the primary antibodies, followed by biotinylated anti-mouse IgG (1:200, 30 minutes; DAKO) and ABC complex (30 minutes; DAKO). Colour was developed with di-aminobenzidine/H_2_0_2_. Double-label immunofluorescence was carried out on the hippocampus and cerebellum using the anti-p62 antibody in combination with anti-TDP-43 or anti-ubiquilin2 antibodies. After appropriate pre-treatment tissue sections were incubated with anti-p62 antibody as described above, followed by a biotinylated secondary antibody (Dako, 1:200), ABC and developed using the TSA Rhodamine kit (Roche). Sections were then incubated with either the anti-TDP-43 or anti-ubiquilin2 antibody followed by Alexa Fluor 488 (Invitrogen, Paisley, UK; 1:300) for one hour at room temperature. 4′-6-diamidino-2-phenylindol (DAPI) was used for nuclear counterstaining. Appropriate controls were used to confirm that no cross reactivity existed between the visualization of the two antibodies. Sections were viewed with a Leica DM5500B fluorescence microscope using 3D deconvolution post-processing.

### Assessment of p62 and TDP-43 pathology

The extent and severity of p62 and TDP-43 positive pathology was evaluated in cases with only a *GRN* mutation or *C9orf72* expansion repeat along with the two cases carrying the double mutations (cases 2.1 and 2.2). A five-tiered semi-quantitative grading scale was used in which the pathological lesions were scored as ‘0’ describing the absence of p62-positive NCIs and NIIs, score ‘+’ corresponded to 1–5 inclusions present in an average of at least five microscopic fields using a ×20 objective, score ‘++’ was given when the number of lesions was 6–10 while score ‘+++’ was given when the number of inclusions was between 11 and 20. Score ‘++++’ corresponded to greater than 20 lesions.

## Results

### Clinical history

**Figure 1:**
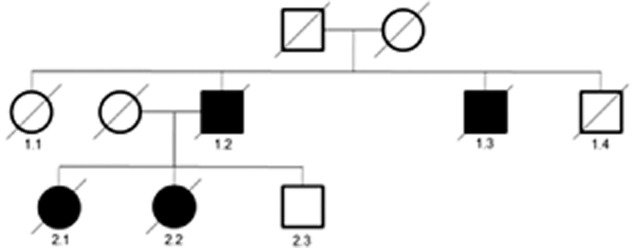
Pedigree of the family. Limited clinical information is available for cases 1.2 and 1.3. Both clinical and pathological data is available for both 2.1 and 2.2.

The DRC 240 family are from the south of the UK and have an autosomal dominant history of speech, cognitive and gait impairment (Figure 1). Little is known about previous generations but case 1.2 was said to have dementia with impaired speech and gait from the age of 61, dying 2 years later whilst case 1.3 also had a young onset dementia with onset at 60 and disease duration of 4 years.

#### Case 2.1

**Figure 2:**
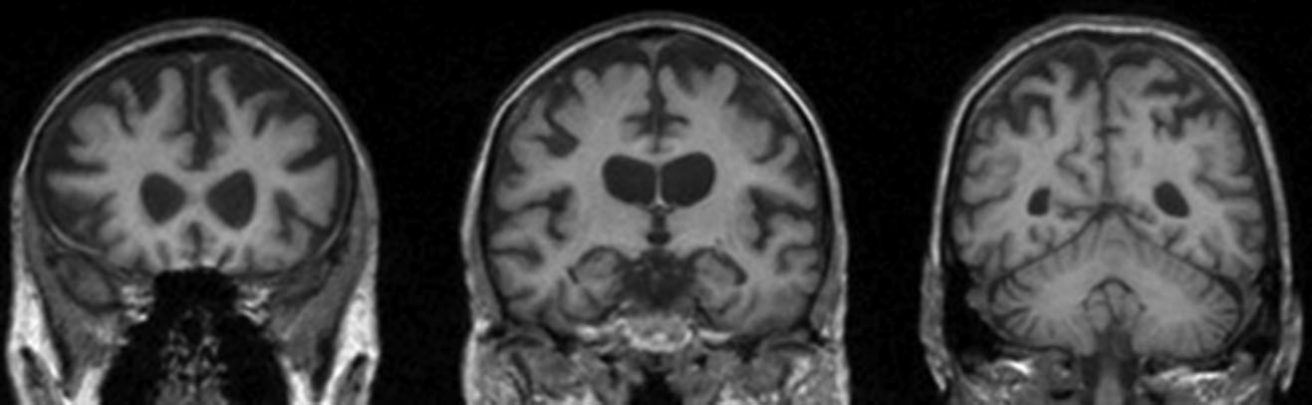
MRI scan of case 2.1. Showing symmetrical atrophy of the hemispheres and enlarged lateral ventricles.

This 62-year-old woman presented with a 3-year history of speech, gait, behavioural and cognitive impairment. Her initial problem was of impaired articulation which steadily deteriorated with subsequent development of an unsteady gait. Over the same time period she had developed a change in her personality, becoming less extrovert than previously and less interested in doing her normal hobbies. In the year before presentation she also developed impairment of her language, spelling and arithmetic skills as well as dysphagia. She had coeliac disease but had been otherwise well previously. On examination her Mini Mental State Examination (MMSE) was 27/30. She had a bulbar dysarthria with decreased palatal elevation and orofacial apraxia. She had mild generalized wasting in the limbs but no fasciculation's. Power was mildly decreased in all muscle groups. Reflexes were brisk throughout with downgoing plantars. She had bilateral dysdiadochokinesis and gait ataxia. Neuropsychometry showed a verbal IQ of 84 and performance IQ of 86. Episodic memory was impaired with difficulties on tasks of naming, spelling, calculation and limb praxis. There was evidence of executive dysfunction but visuoperceptual skills were intact. EMG showed evidence of mild chronic denervation in tibialis anterior bilaterally but was otherwise normal. MRI brain showed relatively symmetrical generalized cortical atrophy with a frontotemporal predominance as well as mild cerebellar atrophy (Figure 2). A diagnosis of probable FTD-MND was made at this time although the features of ataxia were felt to be unusual. She was not seen again and died 4 years later.

#### Case 2.2

This 48-year-old woman presented with a 2-year history of speech, gait and cognitive impairment. She had coeliac disease but had been otherwise well previously. A CT scan showed mild symmetrical generalized atrophy. She died 2 years later.

### Genetics

*GRN* gene sequencing of DNA from both frozen brain and peripheral lymphocytes demonstrated the frameshift mutation *Cys31fs (g.90_91insCTGC)* in exon 2 that results in a null allele reported to be pathogenic [23].

**Figure 3:**
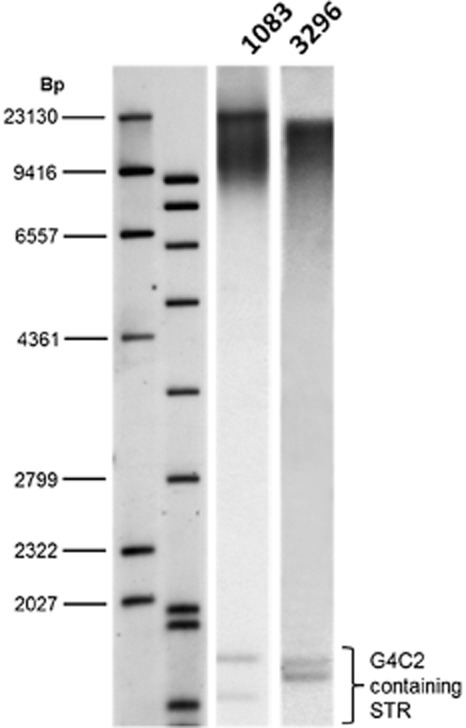
Presence of a heterozygous hexanucleotide expansion in *C9orf72*. Southern blot analysis on DNA using a probe directed to the hexanucleotide repeat (GGGGCC)_5_shows the presence of an expansion in cases 2.1 and 2.2. The presence of a large expansion was confirmed by Southern blotting which revealed a maximum repeat number in the DNA from brain from patient 1 (PDG1083) as 4153 repeats, minimum 1205 (modals measured at 3822, 2500 and 2050 repeats), and in DNA from peripheral lymphocytes from patient 2 (PDG 3296) a maximum of 3316 was measured and minimum of 812 (modal 2780).

rpPCR assessment of *C9orf72* hexonucleotide number demonstrated a minimum of 67 repeats in DNA from both patients on one allele and 2 repeats on the other allele. The presence of a large expansion was confirmed by Southern blotting (Figure 3) which revealed a maximum repeat number in the DNA from brain from case 2.2 (PDG1083) as 4153 repeats, minimum 1205 (modals measured at 3822, 2500 and 2050 repeats), and in DNA from peripheral lymphocytes from case 2.1 (PDG 3296) a maximum of 3316 was measured and minimum of 812 (modal 2780). Genotyping of rs3849942 demonstrated heterozygosity at this polymorphic site for both patients.

### Pathology

#### Macroscopic observations

Cases 2.1 and 2.2 came to post-mortem. The unfixed brain of case 2.1 weighed 1000 g, the left-half brain weighed 452 g after fixation in 10% buffered formaldehyde. There was a significant degree of frontal atrophy, which was also apparent over the medial surface. Coronal slices revealed the left lateral ventricle to be significantly dilated and focal thinning of most of the frontal cortex. There was severe reduction in bulk of the frontal white matter while the white matter of the temporal lobe was relatively better preserved. The lateral aspect of the putamen showed significant grey discolouration, although its bulk was not apparently reduced. However, the size of the caudate was reduced and it had a flattened rather than convex outline towards the left frontal horn. The thalamus was significantly reduced in bulk, whereas the globus pallidus, subthalamic nucleus, amygdala and hippocampus were of normal size. The substantia nigra showed severe pallor, whereas the locus coeruleus was well pigmented. The proportions of the pontine tegmentum and base were preserved and, in particular, the transverse pontine fibres were clearly visible. The medulla, the cerebellar cortex, white matter and dentate nucleus were also of normal appearance. In case 2.2 the right fixed half brain weighed 516 g. Some of the gyri in the frontoparietal region were narrowed and the intervening sulci widened. Coronal slices showed that the right lateral ventricle was moderately enlarged. The cortical ribbon had a blotchy appearance and the border between the cortex and white matter in many places appeared blurred. The hippocampus was macroscopically unremarkable. The substantia nigra and the locus coeruleus were pale. The cerebellum showed no macroscopic abnormality.

#### Microscopic observations

Case 2.1 and 2.2 were both previously diagnosed pathologically as FTLD-TDP type A with *GRN* mutation. Both cases demonstrated TDP-43 NCIs predominantly in layer 2 of the cortex. Numerous small neurites and occasional NIIs were also seen (Figure S1). However, with the identification of p62-positive inclusions in the hippocampus and cerebellum the cases were screened for the *C9orf72* expansion repeat. A detailed pathological review of both cases was undertaken due to the identification of the second pathogenic *C9orf72* mutation.

### Cerebral cortex

Histological slides of the frontal cortex from both cases showed a significant degree of superficial spongiosis, moderate degree of neuronal loss and astrogliosis in the grey matter. TDP-43 immunohistochemistry demonstrated moderate numbers of neurites and TDP-43-positive NCIs. The parietal cortex also showed superficial spongiosis and the presence of TDP-43 positive NCIs were observed. The temporal and occipital cortices also contained the occasional TDP-43 NCI to a lesser extent. p62 immunohistochemistry highlighted the TDP-43 positive inclusions, although a minority of NCI's exhibited a ‘star-like’ shape, which were negative for TDP-43.

### Hippocampal formation

**Figure 4:**
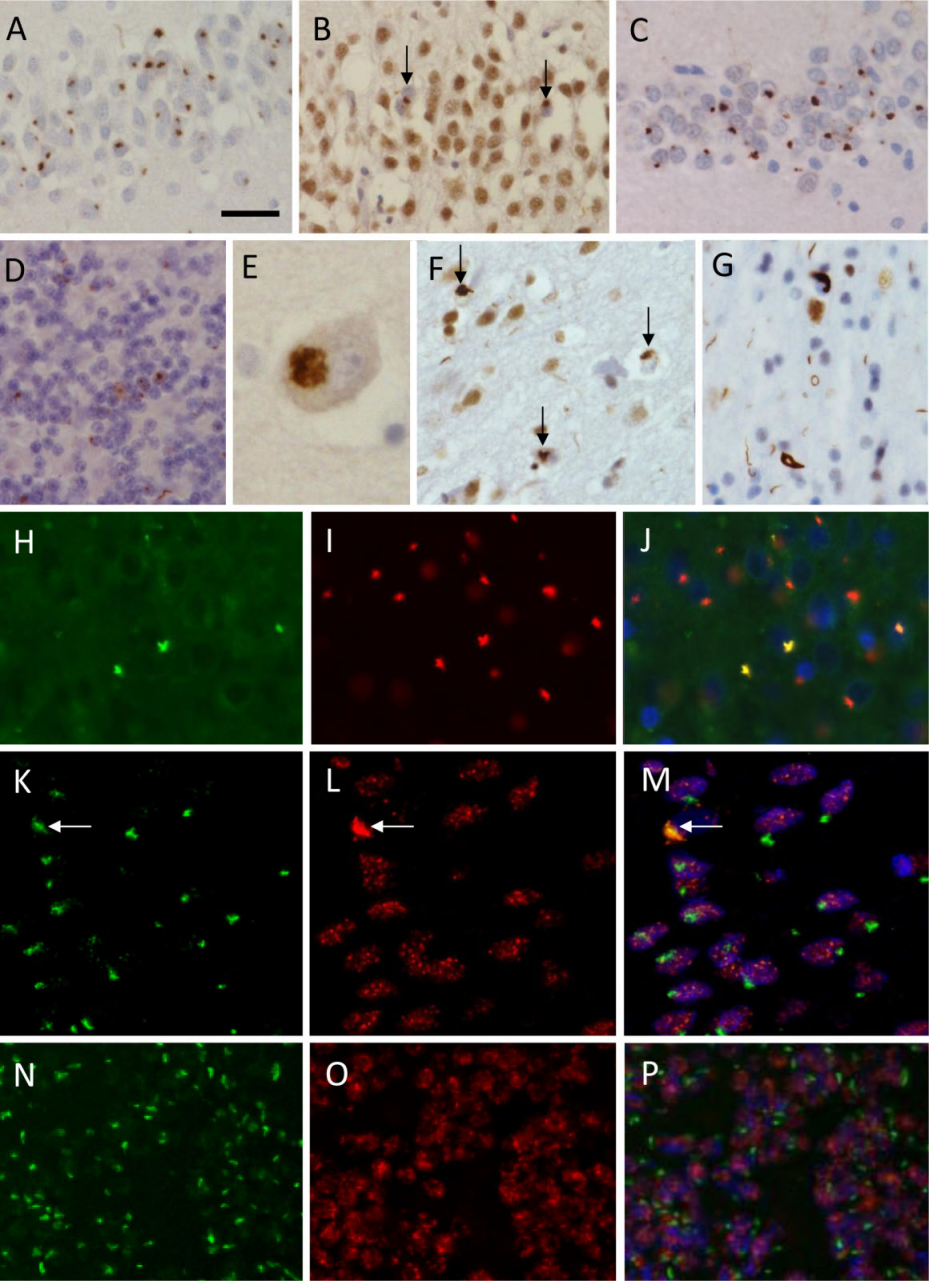
TDP-43, p62 and Ubiquilin2 immunohistochemistry in case 2.1. Numerous p62 positive neuronal cytoplasmic inclusions (NCIs) were evident in the granule cell layer (GCL) of the hippocampus (A) compared to sparse numbers of TDP-43 positive NCIs in the GCL (B, arrows). Ubiquilin2 positive NCIs were also found in the GCL showing similar numbers to those observed with p62 (C). The cerebellum granule cells (D) and the neurones found in other subregions of the hippocampus (CA4) were also found to contain p62-positive ‘star-like’ inclusions (E). TDP-43 pathology was evident in the frontal cortex (F) and found in the frontal cortex white matter (G). Double fluorescent immunohistochemistry revealed that the number of p62 (I) positive inclusions were greater than those immunostained with Ubiquilin2 (H). TDP-43 and p62 immunohistochemistry was combined in the GCL and cerebellum, where a TDP-43 NCI (L, arrow) was outnumbered by the smaller ‘star-like’ p62 positive inclusions (K). This was also seen in the cerebellum where normal nuclear staining of TDP-43 was observed (O) and no colocalization was seen with the p62 positive inclusions (N). Bar on A represents 50 microns on A, B and C; 20 microns on D, F and G; 10 microns on E. The original magnification on panels H-P ×40.

In case 2.1 neurofibrillary tangles (NFTs) and neuropil threads (NTs) were found in the hippocampus, entorhinal and transentorhinal cortices. TDP-43 positive NCIs were observed in the granule cells of the dentate fascia but these were outnumbered by numerous p62-positive ‘star-like’ inclusions (Figure 4). The majority of the p62-positive inclusions were also immunostained with ubiquilin2 (Figure 4), although there were still inclusions that were only positively stained with p62. The p62-positive ‘star-like’ inclusions were also found in the CA4 subregion, which was negative for TDP-43. Case 2.2 showed a similar distribution of TDP-43 and p62 inclusions as that observed in cases 2.1; however no additional pathology was evident.

### Subcortical grey nuclei

The putamen and caudate showed gliosis in case 2.1 confirmed with GFAP immunohistochemistry, where the dorsal regions of these nuclei were more affected than the ventral parts. The subthalamic nucleus was well populated with neurones. Immunostaining for TDP-43 showed numerous neurites in the striatum, some of which had a swollen appearance. NIIs and NCIs, both skein-like and compact were also found. Subcortical grey nuclei were not available for analysis from case 2.2.

### Brainstem

Case 2.1 demonstrated a significant loss of pigmented neurones together with pigment incontinence and gliosis in the substantia nigra, whereas case 2.2 showed only rare extraneuronal pigment. There were no NFTs or Lewy bodies observed in the nigra or other midbrain nuclei in either case. The locus coeruleus was well populated with neurones as were the griseum pontis and other pontine nuclei. The inferior olive showed mild loss of neurones with sparse TDP-43-positive irregular inclusions. No TDP-43 or p62-positive inclusions are identified in the XII^th^ nerve nucleus in case 2.1.

### Cerebellum

The cerebellar cortex in both cases 2.1 and 2.2 showed a moderate degree of Purkinje cell loss and Bergmann gliosis, while the cerebellar dentate nucleus showed a slight depletion of neurones. No TDP-43-positive inclusions were found in any of the cerebellar subregions. However, frequent p62-positive inclusions were found in the cerebellar granule cell layer (Figure 4).

### Additional pathologies

Case 2.1 also demonstrated pathological ageing (Braak and Braak stage II NFT pathology) and cerebrovascular (small vessel) disease, although no Aβ or alpha-synuclein pathology was observed. Whereas in case 2.2 no additional Aβ, tau or alpha-synuclein pathology was observed.

### Clinical comparison with *C9orf72* and *GRN* only cases

The main clinical features in the family are of progressive speech, gait and cognitive impairment. In the case for which most information is known (2.1), a diagnosis of FTD-MND was made with prominent bulbar features. This would be most consistent with an expansion in *C9orf72* although there are rare cases of FTD-MND in association with *GRN* mutations. Interestingly, case 2.1 had cerebellar features clinically, which are not seen in *GRN* mutations, but have been described in some cases of *C9orf72* (Table 1). Case 2.1 had impaired executive function, episodic memory and dominant parietal lobe function – this might be seen in either *GRN* or *C9orf72*, with early episodic memory impairment perhaps more suggestive of *C9orf72*, and early parietal lobe involvement more suggestive of *GRN*, although none of these cognitive features are particularly specific. The brain imaging showed a relatively symmetrical pattern of atrophy (Figure 2), which would be much more in keeping with an expansion in *C9orf72* in comparison to *GRN* mutations where atrophy is asymmetrical. We assessed this in a formal analysis of the volume difference between the right and left hemispheres. In case 2.1, the volume difference between the right and left hemispheres was 10 ml which is not significantly different to a series of fifteen patients with *C9orf72* expansions where the mean (standard deviation) right-left hemisphere volume difference was 13 ml (9 ml), or a group of fifteen age and gender matched cognitively normal controls where the volume difference was 5 ml (5 ml). These were all significantly different to the asymmetrical *GRN* mutation series (10 patients) where the volume difference was 66 ml (30 ml).

**Table 1:** Demographic and clinical data from *GRN/C9orf72* cases compared to *GRN* and *C9orf72* cases

	*C9orf72* Case 1	*C9orf72* Case 2	*C9orf72* Case 3	*C9orf72/GRN* Case 2.1	*C9orf72/GRN* Case 2.2	*GRN* Case 1	*GRN* Case 2	*GRN* Case 3
Demographics								
Gender	Female	Male	Male	Female	Female	Male	Male	Male
Age at onset	68	62	51	59	46	53	56	66
Age at death	75	72	57	66	49	61	64	72
Clinical features								
Main syndrome	FTD-MND	bvFTD	bvFTD	FTD-MND	FTD-MND	bvFTD	bvFTD	bvFTD
Neuropsychiatric and behavioural features								
Disinhibition	+	−	+	−	NK	+	+	+
Apathy	+	+	+	+	NK	−	+	+
Abnormal eating behaviour	+	−	−	−	NK	+	−	+
Obsessive/compulsive behaviour	−	−	−	−	NK	+	−	−
Loss of empathy	−	−	−	−	NK	−	−	+
Cognitive features								
Executive dysfunction	+	NK	+	+	NK	+	NK	+
Language impairment	+	NK	−	+	NK	−	NK	−
Episodic memory impairment	+	NK	+	+	NK	−	NK	+
Parietal lobe dysfunction	−	NK	−	+	NK	+	NK	+
Imaging features								
Symmetry of atrophy (left = L, right = R)	R = L	NK	R = L	R = L	R = L	R > L	NK	R > L

### Pathological comparison with *C9orf72* and *GRN* only cases

The microscopic pathology observed in cases 2.1 and 2.2 was compared with that seen in 3 heterozygous *C9orf72* cases and 3 heterozygous cases carrying only the frameshift *Cys31fs* mutation in exon 2 of *GRN* gene. We compared representative areas of the frontal and temporal cortices along with hippocampal subregions and cerebellum (Table 2). In all *C9orf72* mutation cases along with the TDP-43 positive inclusions, additional p62-positive ‘star-like’ inclusions were found in the granule cells of the dentate fascia, CA4 subregion of the hippocampus and the granule cell layer of the cerebellum. This was evident when the number of TDP-43 and p62 positive inclusions were assessed in a semi-quantitative manner (Table 2). The three *GRN* only cases only demonstrated TDP-43 positive inclusions in all regions and the p62 positive immunohistochemistry mirrored what was observed with the TDP-43. No additional p62-positive star-like inclusions were found in the hippocampus or cerebellum of the *GRN* only cases. In all the *GRN* cases the amount of TDP-43 pathology was increased in the frontal and temporal cortices as well as the number of NCIs observed in the GCL compared to case 2.1 and 2.2.

**Table 2:** Distribution of TDP-43 and p62 positive lesions in *GRN/C9orf72* cases compared to *GRN* and *C9orf72* cases

Anatomical region		Case Number															
C9ORF72 – Case 1		C9ORF72 – Case 2		C9ORF72 – Case 3		Case 2.1		Case 2.2		GRN – Case 1		GRN – Case 2		GRN – Case 3	
TDP	p62	TDP	p62	TDP	p62	TDP	p62	TDP	p62	TDP	p62	TDP	p62	TDP	p62
Frontal Cortex	Grey Matter	++	+	++	++	+++	+++	+	+++	++	++	++++	++	++	++	+++	++
	White Matter	++	++	++	++	++	++	++	+++	++	++	++	+	+	+	++	+
Temporal Cortex	Grey Matter	+	++	+	++	++	+++	+	+++	+	++	+++	+++	+++	+++	+++	+++
	White Matter	+	+	+	+	+	++	++	++	++	++	+	++	++	++	++	++
Hippocampus	GCL	+++	++++	+	+++	++	++++	+	++++	+	++++	++	++	++	++	+++	++
	CA1	+	+++	+	++	0	++++	+	++	0	+++	++	++	0	0	0	0
	CA2	0	+++	0	++	0	+++	0	++	0	++	0	0	0	0	0	0
	CA3	0	+++	0	++	0	+++	0	+++	0	+++	0	0	0	0	0	0
	CA4	0	+++	0	+++	0	+++	0	+++	0	+++	0	0	0	0	0	0
	Subiculum	+	++	+	+	+	++	+	++	+	++	+	+	+	+	0	0
	Entorhinal	+	++	++	++	+	+++	+	++	+	++	++	++	+	+	++	++
	Fusiform	+	++	++	++	++	+++	++	+++	+	+++	++	++	+	+	+++	++
Cerebellar white matter		0	+	0	++	0	+++	0	+	0	+	0	0	0	0	0	0
Cerebellar GCL		0	++	0	++	0	++++	0	++	0	++	0	0	0	0	0	0

Semi-quantitative analysis of TDP-43 and p62 positive lesions observed in three cases carrying the *C9orf72* expansion repeat, three cases carrying a *GRN* mutation and the two family members carrying both the *C9orf72* and *GRN* mutation. Score is an aggregate of all lesions present including neuronal cytoplasmic, neuronal intranuclear inclusions and neurites. Grading: 0 = absent; + = mild; ++ = moderate; +++ = frequent; ++++ = severe. GCL, granule cell layer.

## Discussion

In this study we describe a family that carry both a *GRN* pathogenic mutation and the newly identified *C9orf72* hexanucleotide repeat expansion. Pathologically the two cases were classified as FTLD-TDP type A, with additional p62-positive ‘star-like’ inclusions throughout the different brain regions but predominantly in the hippocampal formation and cerebellum. The family described here were known to segregate for a causal *GRN* mutation. The possibility of concurrence was prompted by the histopathological findings, which were in keeping with those found in *C9orf72* expansion repeat cases. The chance concurrence of the two mutations can be crudely estimated from the population frequencies of the mutations. Pathogenic *GRN* mutations have not been found in the healthy population whereas *C9orf72* mutations are found in approximately 1:700 [24]. As we examined ∼20 *GRN* distantly related cases post-mortem, the probability of a single chance concurrence was approximately 0.03 (20/700). A further possibility is that the concurrence of the mutations increases the penetrance of FTLD; however, in several studies; the penetrance of each mutation in isolation is high. Others have described concurrence of *C9orf72* and TDP-43 mutations [23],[24] and *C9orf72* expansions with 2 novel missense mutations in *GRN (Y294C)* and in *PSEN-2(I146V)* [25]. A systematic study of all causal genes in neurodegeneration will be required to assess whether concurrent causal mutations are found more often than would be expected by chance alone. From a clinical perspective the possibility of concurrent mutations may lead to serious complications when providing genetic testing for affected families. Following the discovery of the link between *GRN* and FTLD, many at-risk family members have come forward for predictive genetic testing, often with influences on major life decisions. The subsequent discovery of a second causal mutation in the family is clearly a tragedy for those having completed such a process and seemingly freed from risk of the family disorder. From a genetic counselling perspective it would therefore be of importance to clarify whether these concurrences are extremely rare and purely chance events, or if they are found more frequently than would be expected because of increased penetrance.

Pathologically both cases demonstrated TDP-43 deposition with numerous NCIs, occasional intanuclear inclusions and short neurites, which are in keeping with the FTLD-TDP type A subtype, previously observed in both *GRN* mutation and *C9orf72* expansion repeat cases. The ‘star-like’ p62 inclusions, found throughout different brain regions, which were readily visible in areas lacking TDP-43 pathology, are characteristic for cases with *C9orf72* expansion repeat. Additional pathology was confirmed in one of our cases in the form of NFT pathology corresponding to Braak and Braak stage II and this case also showed evidence of cerebrovascular disease, which was thought to be ‘age-related’ as this patient was 11 years older at death than the patient without additional pathology.

It remains to be determined how the *C9orf72* expansion repeat length affects the clinical phenotype and the underlying pathology once the expansion repeat can be sized accurately. The two cases analysed in this study demonstrated different repeat lengths, 4153 in case 1 and 3316 in case 2. However, it is of note that in one case brain tissue was used for DNA extraction while peripheral lymphocytes in the other, which may be relevant as it is not known whether the *C9orf72* repeat length is different when it is determined in peripheral tissue or brain tissue.

Both our cases carry two confirmed pathogenic mutations, which raises the question of which of the two genetic abnormalities is driving the underlying pathology or whether both contribute to it. Clinically the main features of this pedigree are of progressive speech, gait and cognitive impairment. A diagnosis of FTD-MND was made in one of the cases, which would be most consistent with an expansion in *C9orf72*, although there are rare cases of FTD-MND in association with *GRN* mutations [25]. Brain imaging showed a symmetrical pattern of atrophy, which would be in keeping with an expansion in *C9orf72,* whereas *GRN* mutations show a more asymmetrical pattern of atrophy [25].

In both of our cases the TDP-43 pathology corresponds to FTLD-TDP type A, which is the pattern seen in cases with *GRN* mutations [26], although cases with *C9orf72* repeat expansion alone can also be associated with this FTLD-TDP subtype [27]. The *Cys31fs GRN* mutation, as identified in the family reported here, is a tetranucleotide insertion in the coding region causing frameshift and premature translation termination, resulting in nonsense-mediated mRNA decay, causing a loss of GRN function rendering the protein less active or ineffective.

Neurologically normal controls have been identified to carry expansion greater than 45 repeats [25]. However, to date, no data from Southern blot analysis are available in control cases, which would provide information about the size of the expansion repeat. Furthermore, in control cases carrying a *C9orf72* repeat expansion no pathological data are available either about whether TDP-43 and/or p62 pathology is present in such cases, which would indicate that they represent a ‘preclinical disease stage’. Therefore without more knowledge of the pathogenic repeat length the *C9orf72* hexanucleotide repeat expansion and data of detailed TDP-43 and p62 immunohistochemical studies in ‘normal’ control cases it would be difficult to suggest the *C9orf72* expansion is merely a risk factor contributing to the susceptibility to the disease in our cases as it has been suggested for another *GRN* mutation case combined with *C9orf72* expansion [28]. However, in that case it remains to be proven if the missense *GRN* mutation is pathogenic or not.

In conclusion, the cases presented here carry ‘double’ pathogenic mutations, both of which have an effect on the cellular pathways finally manifesting in TDP-43 pathology. Future studies are required to understand the precise mechanisms and contribution the two different genetic abnormalities make in such cases.

## Supporting Information

Figure S1 TDP-43 pathology in GRN/C9orf72 cases. Cases 2.1 and 2.2 both demonstrated FTLD-TDP type A pathology. Neuronal cytoplasmic inclusions were found in the granule cell layer of the hippocampal formation (A, arrow). Occasional neuronal intranuclear inclusions were found in the frontal cortex (B, arrow) together with neuronal cytoplasmic inclusions mainly found in layer two of the cortex (C). Many short dystrophic neurites were also found (D).
