# Supplementary figures and images for: A pathogenic progranulin mutation and C9orf72 repeat expansion in a family with frontotemporal dementia
Source: Neuropathol Appl Neurobiol. 2014 Apr 22;40(4):502–13. doi: 10.1111/nan.12100 (PMC4260146; doi:10.1111/nan.12100)

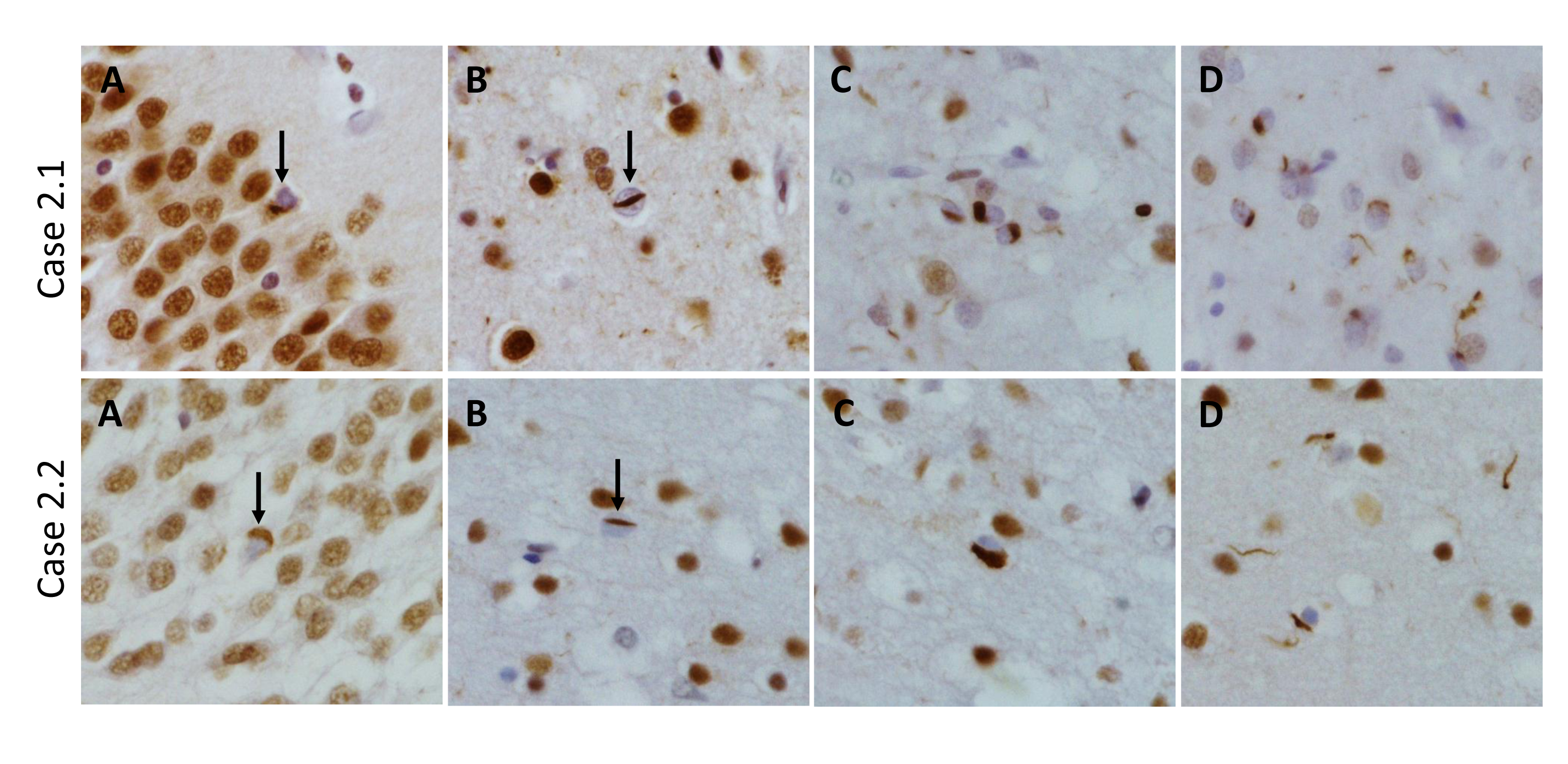

Supplement: Figure S1 — TDP-43 pathology in GRN/C9orf72 cases. Cases 2.1 and 2.2 both demonstrated FTLD-TDP type A pathology. Neuronal cytoplasmic inclusions were found in the granule cell layer of the hippocampal formation (A, arrow). Occasional neuronal intranuclear inclusions were found in the frontal cortex (B, arrow) together with neuronal cytoplasmic inclusions mainly found in layer two of the cortex (C). Many short dystrophic neurites were also found (D). [file nan0040-0502-SD1.tif]
